# Correction to ‘Early experience of Sacubitril–Valsartan in heart failure with reduced ejection fraction in real‐world clinical setting’

**DOI:** 10.1002/ehf2.15211

**Published:** 2025-01-17

Nordberg Backelin, C. , Fu, M. , and Ljungman, C. (2020) Early experience of Sacubitril–Valsartan in heart failure with reduced ejection fraction in real‐world clinical setting. ESC Heart Failure, 7: 1049–1055. 10.1002/ehf2.12644.32030899 PMC7261574

In the abstract, in the last sentence of methods and results, the text ‘Female gender [odds ratio (OR) 3.58; 95% CI 1.07–2.00; *P* = 0.038] and NT‐proBNP ≥ median level (OR 0.48; 95% CI 0.26–0.90; *P* = 0.021) was associated with termination of the medication’ was incorrect. This should have read ‘Female gender [odds ratio (OR) 3.58; 95% CI 1.07–2.00; *P* = 0.038] was associated with termination of the medication’.

Also, in page 1051, in the last paragraph of the results, the text ‘Age‐adjusted logistic regression analysis showed that female gender (OR 3.58; 95% CI 1.07–2.00; *P* = 0.038) and a NT‐proBNP higher than the median level of 2,860 ng/L (OR 0.48; 95% CI 0.26–0.90; *P* = 0.021) predicted discontinuation of the treatment’ was wrong. This should have read ‘Age‐adjusted logistic regression analysis showed that female gender (OR 3.58; 95% CI 1.07–2.00; *P* = 0.038) predicted discontinuation of the treatment’.

We apologize for this error.

